# Supplementary material for: Mechanisms governing the pioneering and redistribution capabilities of the non-classical pioneer PU.1
Source: Nat Commun. 2020 Jan 21;11:402. doi: 10.1038/s41467-019-13960-2 (PMC6972792; doi:10.1038/s41467-019-13960-2)
Supplement: Supplementary file 7 — Source data [file 41467_2019_13960_MOESM7_ESM.zip › Source_Data/FigureS2/FigureS2B_MotifScanOutput/KmeansCluster2/homerResults/motif26.similar.html]

motif26

## Information for motif26

A
G
T
C
T
C
G
A
G
A
C
T
C
T
A
G
T
C
G
A
C
A
G
T
A
G
T
C
A
G
T
C
T
C
G
A
T
C
G
A
C
G
A
T
A
G
T
C
C
G
T
A
A
G
T
C
  
Reverse Opposite:  

C
T
A
G
C
G
A
T
A
C
T
G
C
G
T
A
A
G
C
T
A
C
G
T
C
T
A
G
T
C
A
G
G
C
T
A
A
C
G
T
G
A
T
C
C
G
T
A
A
C
G
T
C
T
A
G
  

|  |  |
| --- | --- |
| p-value: | 1e-46 |
| log p-value: | -1.066e+02 |
| Information Content per bp: | 1.888 |
| Number of Target Sequences with motif | 70.0 |
| Percentage of Target Sequences with motif | 1.39% |
| Number of Background Sequences with motif | 24.8 |
| Percentage of Background Sequences with motif | 0.06% |
| Average Position of motif in Targets | 102.8 +/- 79.0bp |
| Average Position of motif in Background | 111.2 +/- 55.8bp |
| Strand Bias (log2 ratio + to - strand density) | 0.1 |
| Multiplicity (# of sites on avg that occur together) | 1.00 |
| Motif File: | file (matrix) reverse opposite |

### Similar de novo motifs found

|  |  |  |  |  |  |  |  |
| --- | --- | --- | --- | --- | --- | --- | --- |
| Rank | Match Score | Redundant Motif | P-value | log P-value | % of Targets | % of Background | Motif file |
| 1 | 0.943 | G T C A C G A T C T A G C T G A C A G T A T G C A G T C T G C A T C G A G C A T G T A C G C T A A G T C | 1e-43 | -100.500647 | 1.41% | 0.08% | motif file (matrix) |
| 2 | 0.920 | A G T C T C G A G A C T C T A G T C G A C A G T A G T C A G T C T C G A C T G A A C G T G T A C | 1e-40 | -93.454075 | 1.45% | 0.11% | motif file (matrix) |
